# Diamond Metasurface‐Based Optical Tweezers With Enhanced Robustness

**DOI:** 10.1002/advs.202524086

**Published:** 2026-02-04

**Authors:** Jing‐Yuan Zhu, Ke‐Xue Li, Pei‐Nan Ni, Shang‐Heng Li, Si‐Rui Wang, Wen‐Jie Dou, Yong‐Yang Zhu, Zhi‐Peng Wei, Chong‐Xin Shan

**Affiliations:** ^1^ Henan Key Laboratory of Diamond Materials and Devices Key Laboratory of Materials Physics Ministry of Education School of Physics Zhengzhou University Zhengzhou China; ^2^ State Key Laboratory of High‐Power Semiconductor Laser State Key Laboratory of High‐Power Semiconductor Lasers School of Physics Changchun University of Science and Technology Changchun China

**Keywords:** diamond, lab‐on‐a‐chip, metasurfaces, optical tweezers, thermal robustness

## Abstract

Optical tweezers have revolutionized the manipulation of micro‐ and nano‐scale particles, with impacts across biophysics, materials science, and quantum optics. However, their miniaturization for lab‐on‐a‐chip applications is hindered by bulky optical components. While metasurface‐based optical tweezers offer an ultracompact alternative, they suffer from laser‐induced thermal effects, which degrade their performance, stability, and durability. Here, we overcome this challenge with diamond metasurfaces, leveraging the material's exceptional thermal conductivity, low thermal expansion, and high optical damage threshold to ensure structural integrity under high‐power illumination. We experimentally demonstrate versatile particle manipulations using diamond metasurface optical tweezers, including 2D trapping, precise translocation, and controlled rotation via angular momentum transfer. This work not only resolves the critical thermal limitations of conventional metasurface optical tweezers but also establishes a robust platform for high‐power, miniaturized optomechanical systems, paving the way for their scalable integration into demanding photonic applications.

## Graphical abstract

Conventional metasurface optical tweezers are hindered by thermal instability from laser heating. This work overcomes this by using diamond metasurfaces, exploiting their low thermo‐optic coefficient and minimal thermal expansion. The platform demonstrates stable particle trapping, translocation, and rotation, enabling robust miniaturized systems for advanced photonic and optomechanical applications.

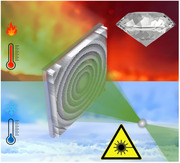

## 1 Introduction

Optical tweezers use highly focused laser beams for the noninvasive optomechanical manipulation of micro‐ and nano‐scale particles, enabling control over targets from whole cells to individual molecules [1, 2, 3, 4]. This technique has significantly expanded the frontiers of scientific research across numerous disciplines. For example, optical tweezers have become indispensable for conducting high‐precision measurements of piconewton‐scale forces and nanometre‐level displacements, enabling foundational studies in molecular motors, protein folding, DNA mechanics, and cellular interactions [5, 6, 7, 8, 9]. These applications have profoundly advanced fields such as biophysics and single‐molecule biology. Furthermore, optical tweezers play a critical role in colloidal science for assembling nanostructures and in quantum optics for manipulating individual atoms or probing quantum coherent systems [10, 11, 12, 13].

The rapid advancement of lab‐on‐a‐chip technology, which integrates multiple laboratory functions onto a single chip, requires the development of ultracompact optical tweezers [14, 15]. However, conventional systems typically rely on the use of bulky and expensive optical elements, such as spatial light modulators (SLMs) [16] and high‐numerical‐aperture objectives [17], which prevents its miniaturization and integration. Over the past decade, metasurfaces, composed of engineered 2D nanoantenna arrays capable of subwavelength light manipulation, have emerged as an ultrathin, lightweight alternative to conventional optics [18, 19, 20, 21, 22, 23, 24, 25]. This technique offers superior resolution and versatile optical functions, holding significant potential for miniaturized optical tweezers capable of advanced multifunctional optical manipulation [26, 27]. However, metasurface optical tweezers operate by concentrating laser energy using subwavelength nanostructures to generate high optical gradients, which inevitably causes localized heating due to optical absorption. This issue is especially pronounced in metallic metasurfaces, where ohmic and interband losses lead to significant temperature increases. Even all‐dielectric metasurfaces suffer from non‐negligible absorption at high laser intensity, especially near engineered resonant modes. Therefore, effective thermal management poses a critical challenge in metasurface optical tweezers, necessitating strategic solutions to ensure reliable device operation and structural integrity, particularly under high‐power or continuous‐wave laser illumination.

Diamond features a unique combination of properties that make it an ideal material for metasurfaces, especially in applications demanding efficient thermal management. In particular, diamond exhibits the highest thermal conductivity (> 2000 W/m·K) among all natural materials, enabling rapid and uniform dissipation of heat away from localized hot spots [28, 29]. Moreover, owing to its low thermal expansion coefficient, diamond maintains exceptional dimensional stability under thermal stress, preventing structural deformation and minimizing optical performance drift even under high‐power laser irradiation. Furthermore, diamond demonstrates a high optical damage threshold, allowing it to withstand intense laser illumination without degradation, thereby supporting stable operation at higher power levels. Based on these advantages, diamond metasurface represents a promising platform for next‐generation optical tweezers, combining precise wavefront control with unparalleled thermal management capabilities. However, diamond metasurface optical tweezers have not been demonstrated previously.

In this work, we present the experimental demonstration of versatile optical tweezers using diamond metasurfaces that enable sophisticated motional control of microspheres, including stable 2D trapping, precise translocation, and controlled rotation via angular momentum transfer. This approach provides a robust solution to the critical challenge of thermal degradation in conventional metasurface optical tweezers. Consequently, our work establishes a new paradigm for high‐power, stable optomechanical manipulation and represents an important advancement toward practical, scalable integrated photonic systems for applications in biomedical engineering, quantum technologies, and beyond.

## 2 Results and Discussion

**FIGURE 1:**
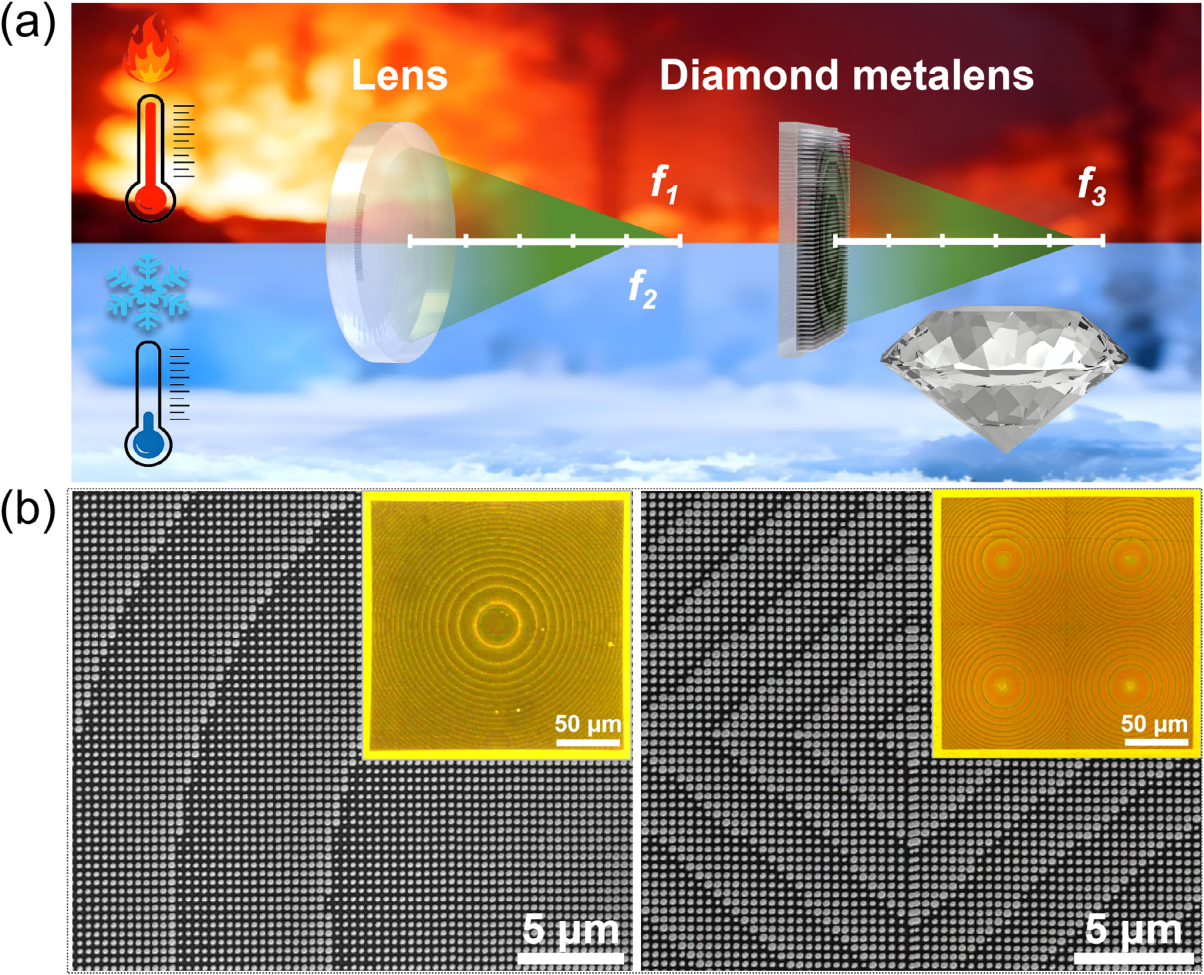
(a) Schematic comparison of focal length variations under dramatic temperature changes, the diamond metalens shows a negligible focal shift, while a conventional lens experiences substantial focal drift. (b) Scanning electron microscopy (SEM) and optical microscopy images of the fabricated single‐element diamond metalens (left) and 2 × 2 diamond metalens array (right).

Optical tweezers provide a high‐precision tool for the non‐contact manipulation of micro‐ and nano‐scale particles through the exertion of optical forces arising from strongly focused laser beams. These forces can be mainly classified into gradient and scattering components, which collectively form an optical potential well near the focal region [30]. Within this region, dielectric particles with a refractive index higher than the surrounding medium are confined at the position of maximum electric field intensity [31]. The physical basis of this trapping mechanism lies in the transfer of photon momentum, resulting in controlled mechanical motion of the target particles. Moreover, through the coordinated interplay of these optical forces, complex trajectory control of microspheres can be achieved with high spatial and temporal precision. However, conventional optical lenses suffer from thermally induced focal shifts under varying environmental conditions due to their substantial coefficients of thermal expansion (CTE) and thermo‐optic coefficient (d*n*/d*T*). In contrast, owing to diamond's exceptionally low CTE and near‐zero d*n*/d*T* (See Table S1), diamond metalenses exhibit exceptional thermal stability, maintaining a nearly invariant focal length across a broad temperature range, as illustrated in Figure 1a. This inherent thermal robustness renders them particularly suitable for applications in environments with large thermal fluctuations or under prolonged high‐power laser illumination. To experimentally investigate the thermal stability of diamond metalenses, a single metalens (left panel, Figure 1b) and a 2 × 2 metalens array (right panel, Figure 1b) were designed and fabricated, operating at wavelengths of 1064 and 532 nm, respectively. (See Notes S1 and S2 for details).

**FIGURE 2:**
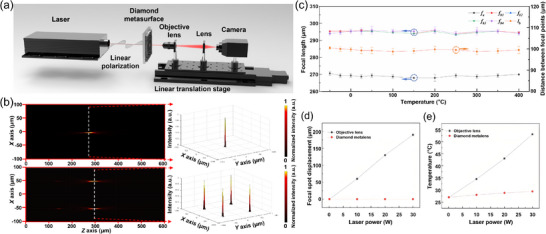
(a) Schematic diagram of the experimental setup for characterizing the focusing performance of diamond metalenses. (b) Measured focusing characteristics of both single‐element and 2 × 2 diamond metalenses at room temperature. (c) Thermal stability evaluation: focal length variation of the single‐element metalens (denoted as *f_a_*) and the four focal lengths of the 2 × 2 metalens array (denoted as *f_b_*_1_, *f_b_*_2_, *f_b_*_3_, and *f_b_*_4_) across different temperatures, with the right *y*‐axis showing the average inter‐spot distance of the quad‐focus metalens array (denoted as *l*_b_) as a metric for positional stability. (d) Comparison of focal spot displacement between a conventional objective lens and the diamond metalens under increasing laser power. (e) Corresponding temperature rise of the conventional objective lens vs. the diamond metalens under the same laser power conditions.

The focusing performance of the diamond metasurfaces under varying temperatures was characterized using a custom optical system (Figure 2a). The setup employed continuous‐wave (CW) lasers as excitation sources, a high‐numerical‐aperture objective (60×, NA = 0.85), and a motorized translation stage for axial scanning. The focal intensity was recorded by a CCD camera via an imaging lens. The microscope objective, imaging lens, and CCD were mounted on a common platform to ensure a stable and reproducible optical path. Following precise alignment of all components, the diamond metalens was securely positioned on a translation stage with its backside normal to the incident beam direction, defining the metasurface plane as *Z* = 0 µm. To evaluate the focal properties of the diamond metalens, transverse beam profiles (*X–Y* plane) were recorded at incremental axial positions along the propagation direction (*Z*‐axis) by capturing the spatial intensity distribution via the CCD. For the 2 × 2 metalens array, the same procedure was repeated using a 532 nm CW laser as the excitation source. Figure 2b presents the optical characterization results for both the single‐element diamond metalens (top panel) and the 2 × 2 diamond metalens array (bottom panel), measured at room temperature. The left image in each panel displays the measured intensity distribution in the longitudinal cross‐section (*X‐Z* plane). The single‐element metalens exhibits a focal length of approximately 270 µm, while the 2 × 2 metalens array has a focal length of approximately 300 µm. The right image in each panel displays the reconstructed 3D intensity profile. It can be seen that the single‐element metalens produces a well‐defined focal spot with a full‐width at half‐maximum (FWHM) diameter of approximately 3 µm. In the case of the 2 × 2 metalens array, four distinct and uniformly spaced focal spots are clearly resolved in a square arrangement at the focal plane, with an inter‐spot distance of approximately 100 µm and individual spot diameters consistently around 3 µm, confirming high uniformity and diffraction‐limited performance. Furthermore, the focusing performance of both the single‐element diamond metalens and the 2 × 2 diamond metalens array was systematically characterized across a broad temperature range from –50°C to 400°C. As summarized in Figure 2c, all the fabricated devices maintained highly consistent focal properties under thermal variations. The right *Y*‐axis indicates the average inter‐spot distance within the metalens array, which serves as a metric for evaluating lateral positional stability under thermal perturbation. Note that all measurements were performed under steady‐state conditions after thermal equilibrium was reached at each target temperature. The results indicate negligible drift in both focal length and relative spot alignment over the entire temperature range, confirming the outstanding thermal stability of diamond metasurfaces and their potential for deployment in environments with severe thermal fluctuations. We further evaluated the focal stability of the diamond metalens under high‐power laser irradiation. As shown in Figure 2d,e, the diamond metalens maintained a stable focal position and exhibited only a minimal temperature rise across incident powers from 0.1 to 30 W. In contrast, a conventional objective lens suffered a substantial focal shift of ∼200 µm and significantly greater heating under the same conditions. These results directly demonstrate the superior thermal and structural stability of the diamond metalens for high‐power applications. Moreover, a critical limitation of the conventional objective lens is its severe focal drift under prolonged high‐power operation (e.g., 30 W), which destabilizes optical traps. The diamond metalens demonstrated remarkable robust performance, showing almost no observable drift under the same laser power and over an even longer duration (Figure S4). Furthermore, the focusing efficiency of the diamond metalens proved highly robust, showing negligible change over the tested ranges of laser power and temperature (Figure S5).

**FIGURE 3:**
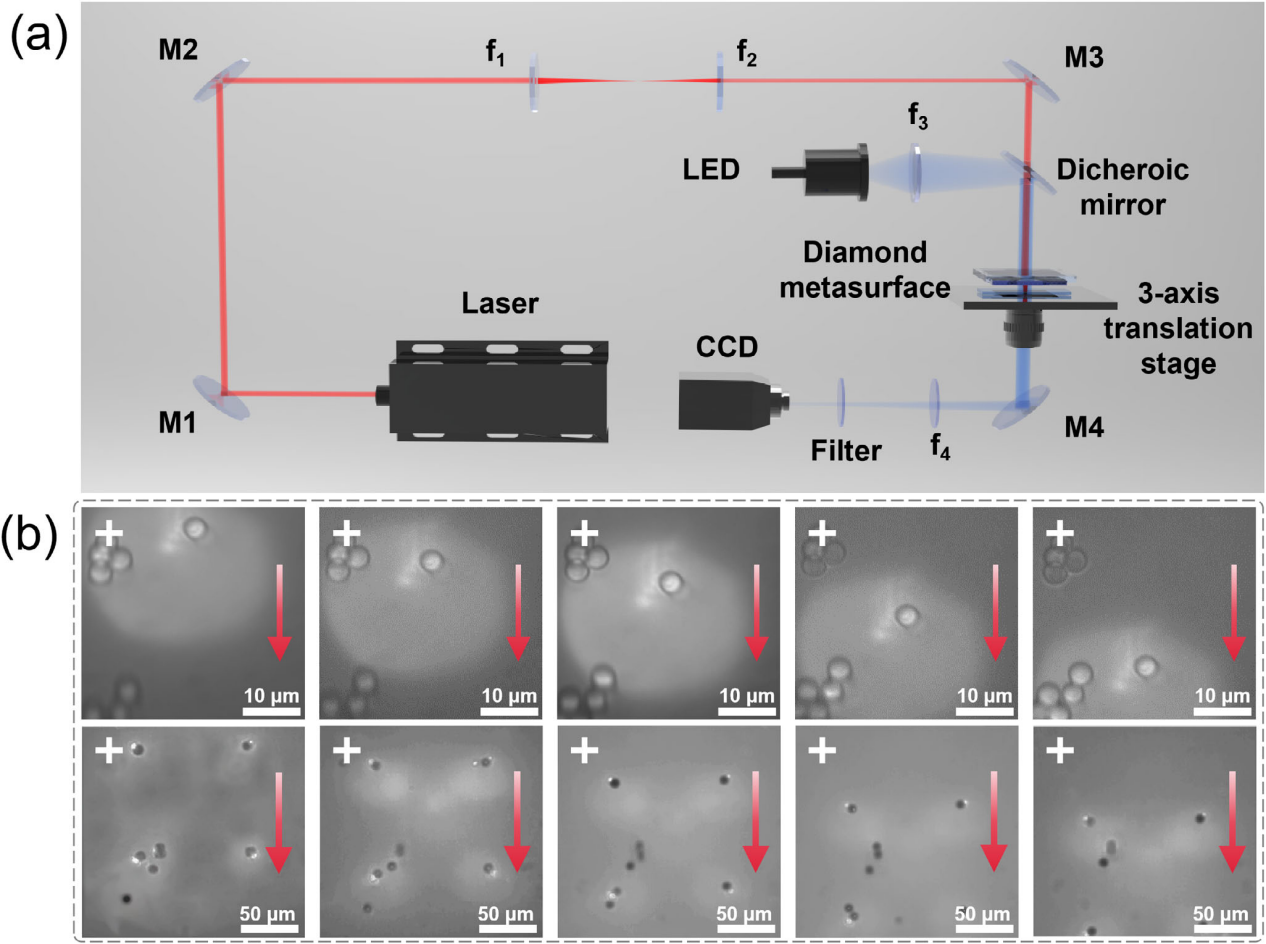
(a) Schematic of the optical tweezer's setup incorporating a metasurface as the wavefront‐shaping element. (b) Demonstration of stable optical trapping and controlled translocation of silica microspheres using a single‐element diamond metalens (top) and a 2 × 2 metalens array (bottom), respectively, confirming precise multiparticle manipulation capabilities.

The capability of the diamond metalens for optical trapping and translocation was demonstrated with a system incorporating both a single‐element metalens and a 2 × 2 metalens array (Figure 3a, see Experimental Methods for details). In this configuration, the diamond metalens was mounted on a motorized coaxial *X*‐*Y* translation stage to enable precise axial alignment, while a sample dispersed with silica microspheres was positioned on a three‐axis translation stage. This arrangement facilitated controlled optical trapping and translocation of individual microsphere. Under illumination with a 1064 nm CW laser, a single silica microsphere was stably trapped at the focal point. Following successful trapping, the microsphere was transported to a predefined location using the three‐axis translation stage. Figure 3b summarizes this process, with a red arrow indicating the direction of motion and a white cross marking a fixed reference position across successive images. Furthermore, simultaneous manipulation of four silica microspheres was achieved by integrating the 2 × 2 diamond metalens array into the optical tweezer system illuminated by a 532 nm CW laser. It can be seen that all four microspheres were translated downward synchronously while maintaining a constant inter‐particle spacing of approximately 100 µm, demonstrating stable multi‐particle manipulation capability. Videos of microspheres transfer between the traps are further provided as Videos S1 and S2, respectively.

Note that the numerical aperture (NA) of a single‐element diamond metalens is approximately 0.35, calculated using the formula NA ≈ n*sin(arctan(*D*/(2*f*)), where *n* is the refractive index of the immersion medium (air, *n* = 1), *D* is the lens diameter, and *f* is the focal length. This moderate NA was chosen to enable a larger field of view and extended working distance, which supports parallel manipulation and system integration in a compact platform. As a result, the optical trapping demonstrated here is primarily 2D, with particles stably confined in the lateral (*X*‐*Y*) plane. Furthermore, it is important to emphasize that the multifunctional capabilities of metasurfaces significantly expand the toolkit available for optical trapping and manipulation. For example, controlled rotation of microparticles can be achieved using metasurfaces designed to impart orbital angular momentum (OAM) from an optical vortex beam to the target particles [32, 33]. The vortex beam features a helical wavefront described by the phase term exp(*i* *l* *ϕ*), where *l* is the topological charge and *ϕ* the azimuthal angle [34]. This phase structure produces a characteristic annular intensity profile with a central phase singularity. When a microparticle is trapped within the high‐intensity ring of the vortex beam, it experiences asymmetric optical forces due to the spatially varying phase gradient. The transfer of orbital angular momentum from the photons to the particle induces a torque, resulting in rotation around the optical axis. The magnitude of this torque and the resulting rotational frequency depend on both the topological charge *l* and the incident laser power [17].

**FIGURE 4:**
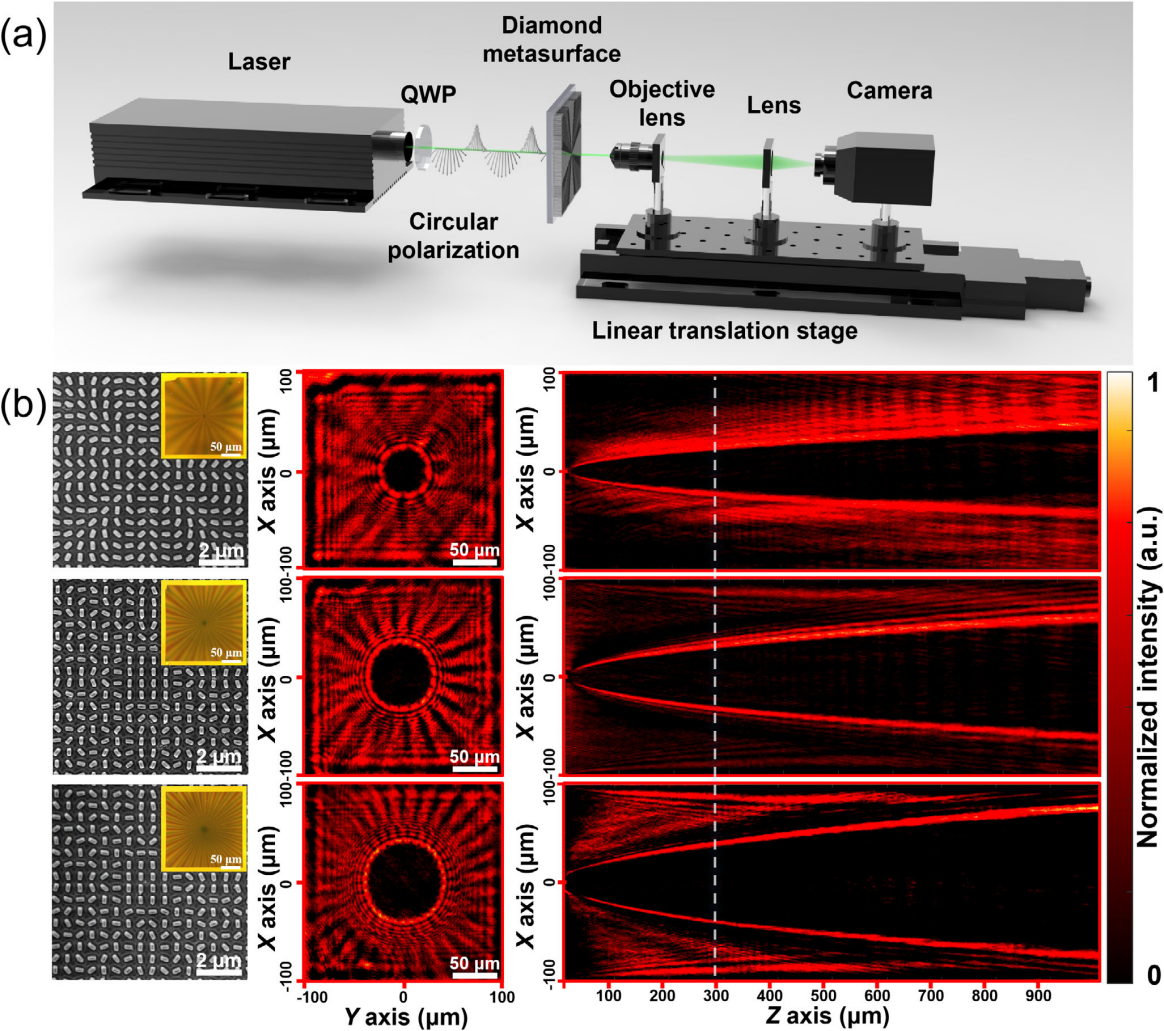
(a) Schematic illustration of the experimental setup for characterizing the orbital angular momentum (OAM) generation and beam‐shaping performance of diamond vortex metasurfaces. (b) Performance evaluation of diamond vortex metasurfaces with varying topological charges (*l* = 10, 20, 30), including transverse beam profiles (*X*–*Y* plane) at *Z* = 300 µm and the reconstructed beam propagation profile in the *X*–*Z* plane. The white dashed line in the right panel indicates the plane at *Z* = 300 µm.

To validate this functionality experimentally, three distinct diamond vortex metasurfaces with topological charges *l* = 10, 20, and 30 were designed and fabricated based on the geometric Pancharatnam–Berry (PB) phase principle (See Note S3 for details). Figure 4a illustrates the experimental setup used to characterize the performance of these diamond metasurfaces. A 532 nm CW laser serves as the illumination source, with a quarter‐wave plate converting linearly polarized light into circular polarization. A CCD camera captures the spatial intensity profiles of the transmitted beam. The microscope objective, relay lenses, and camera are mounted on a motorized translation stage to enable scanning along the propagation axis (*Z*‐direction) for analysis of the beam properties after transmission through the vortex metasurfaces. The metasurface plane is defined as *Z* = 0 µm, with laser illumination occurring normal to the substrate. The left panel in Figure 4b shows the SEM and optical images of the fabricated diamond vortex metasurface with* l* = 10, 20, and 30. The center panel displays the measured transverse intensity distribution (*X*–*Y* plane) at *Z* = 300 µm. The right panel shows the reconstructed beam propagation profile in the *X*–*Z* plane. The results confirm that all three metasurfaces generate vortex beams exhibiting the expected annular intensity distribution with a central dark core. Furthermore, we observe a monotonic increase in the central singularity's diameter with topological charge, in agreement with theoretical predictions.

**FIGURE 5:**
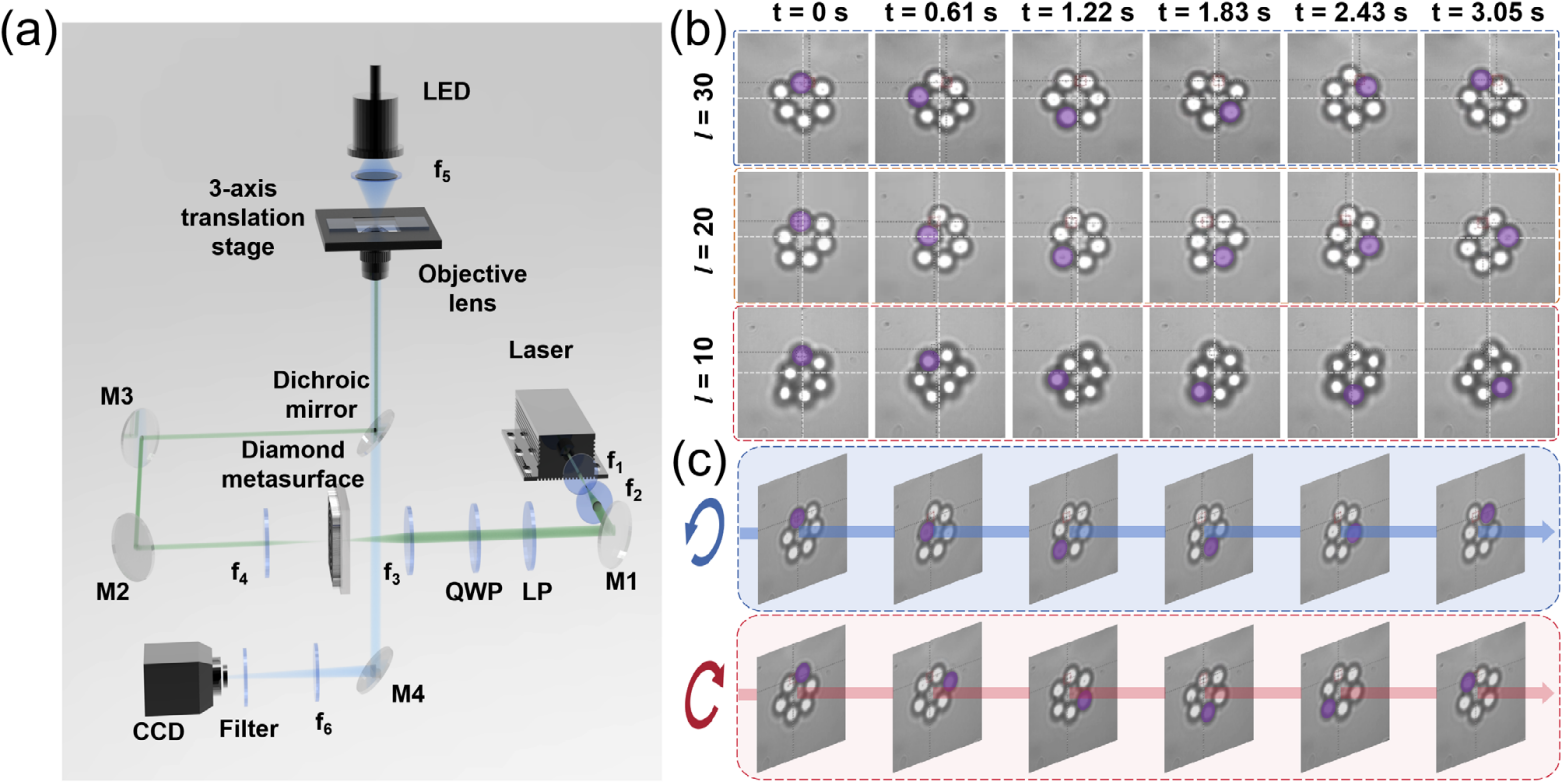
(a) Schematic of the optical tweezer's setup employing a diamond vortex metasurface for controlled rotational manipulation of silica microspheres. (b) Measured rotation speeds of trapped microspheres under vortex beams with topological charges of *l* = 10, 20, and 30, showing a monotonic increase in rotation speed with increasing topological charge. (c) Bidirectional rotation control demonstrated by switching the topological charge from +20 to −20 through reversal of the handedness of the incident CP light, resulting in a corresponding switch from clockwise to counterclockwise rotation.

Figure 5a schematically illustrates the optical tweezer system designed for rotational manipulation of microspheres using a diamond vortex metasurface. A 532 nm CW laser was employed as the excitation source, which was collimated and then expanded to minimize divergence and ensure a uniform power density profile. A linear polarizer combined with a quarter‐wave plate converted the linear polarization to circular polarization. The diamond vortex metasurface was mounted on a co‐axial *XY* translation stage to facilitate precise alignment. The resulting optical vortex beam was directed through a 650 nm long‐pass dichroic mirror and subsequently focused by the objective lens. To quantify the influence of the topological charge on the orbital rotation speed of optically trapped silica microspheres, vortex beams with topological charges of *l* = 10, 20, and 30 were generated using diamond metasurfaces under a fixed incident laser power of 1 W. For fair comparison, all experimental parameters were maintained constant except for the topological charge value of the diamond vortex metasurface. The motion of the trapped particles was recorded with a CCD camera at a sampling interval of Δ*t* = 0.61 s, with six consecutive frames captured for each topological charge. As summarized in Figure 5b, the microspheres underwent stable orbital rotation in the clockwise direction, with measured rotation speeds of approximately 0.20 rps for *l* = 10, 0.28 rps for *l* = 20, and 0.34 rps for *l* = 30, respectively (Video S3). These results clearly demonstrate a monotonic increase in rotation speed with increasing topological charge. Furthermore, bidirectional rotational control (clockwise and counterclockwise) of trapped particles can be achieved using the same diamond vortex metasurfaces by switching the handedness of the incident circularly polarized light. Specifically, the metasurface designed based on the PB phase principle converts LCP light into a vortex beam carrying a topological charge of +20, and RCP light into a vortex beam with *l* = −20, thereby inducing opposite rotational directions (Video S4). Representative trajectories extracted from six images captured at 0.61s intervals under each condition are highlighted in purple in Figure 5c. Comparative analysis indicated no statistically significant difference in rotation speed between the two directions, confirming that the direction of rotation is determined solely by the sign of the OAM. This ability to precisely control rotational direction via OAM sign reversal holds considerable potential for applications in lab‐on‐a‐chip systems and optically driven micromachines.

## 3 Conclusion

In summary, in this work, we established a robust and versatile platform for optical tweezers based on diamond metasurfaces, representing a significant advance in micro‐and nano‐optical manipulation under demanding operational conditions. Capitalizing on diamond's exceptional properties, including its ultrahigh thermal conductivity, high optical damage threshold, and low coefficient of thermal expansion, diamond metasurfaces exhibit inherent thermal stability and maintain structural robustness even under abrupt environmental temperature fluctuations or prolonged high‐power laser illumination. This capability enables sustained and reliable device operation in regimes where conventional metasurfaces would suffer from rapid thermal degradation. Experimentally, we demonstrate versatile particle control using diamond metasurfaces optical tweezers, achieving multifunctionalities, such as stable 2D trapping, precise translocation, and controlled rotation via angular momentum transfer. These functionalities highlight the system's compact footprint and integrated versatility. By providing a thermally stable, high‐power, and ultracompact manipulation platform, this work paves the way for next‐generation optomechanical systems, with promising applications in high‐throughput lab‐on‐a‐chip system, precision nanofabrication, and quantum optomechanics, where stringent thermal management and miniaturization are essential.

## 4 Experimental Methods

### 4.1 Construction of the Optical Tweezer Systems Using Diamond Metalenses

The silica microsphere solution (SiO_2_, 4 µm diameter) was diluted with deionized water at a volume ratio of 1:10, and the resulting solution was encapsulated in a sealed thin fluid chamber constructed from glass slides and cover slips to prepare experimental samples for subsequent use. A CW laser source (1064 nm, 1000 mW) was directed through mirror M1 into a beam compressor composed of two lenses (*f*_1_ = 200 mm and *f*_2_ = 40 mm) to match the metasurface dimensions. The collimated beam was then reflected by mirror M2, directed through a dichroic mirror, and focused by the diamond metalens to manipulate the SiO_2_ microspheres. The imaging system utilized a white LED (GCI‐060411) with a lens as an illumination source. An objective lens was positioned beneath the sample for real‐time imaging, and the video image was subsequently relayed via mirror M3 through a lens‐filter assembly to a CCD camera for observation. Real‐time tracking of microsphere motion was achieved using a scientific‐grade CCD camera equipped with a 950 nm short‐pass filter (MEFH10‐950SP) and a lens.

## Conflicts of Interest

The authors declare no conflicts of interest.

## Supporting information




**Supporting File 1**: advs74064‐sup‐0001‐SuppMat.docx.

**Supporting File 2**: advs74064‐sup‐0002‐VideoS1.mp4.

**Supporting File 3**: advs74064‐sup‐0003‐VideoS2.mp4.

**Supporting File 4**: advs74064‐sup‐0004‐VideoS3.mp4.

**Supporting File 5**: advs74064‐sup‐0005‐VideoS4.mp4.

## Data Availability

The data that support the findings of this study are available from the corresponding author upon reasonable request.
