# Supplementary material for: Diamond Metasurface‐Based Optical Tweezers With Enhanced Robustness
Source: Adv Sci (Weinh). 2026 Feb 4;13(19):e24086. doi: 10.1002/advs.202524086 (PMC13045361; doi:10.1002/advs.202524086)
Supplement: Supplementary file 1 — Supporting File 1: advs74064‐sup‐0001‐SuppMat.docx. [file ADVS-13-e24086-s004.docx]

**Supplementary Information for**

**Diamond Metasurface-Based Optical Tweezers with Enhanced Robustness**

Jing-Yuan Zhu^1,‡^, Ke-Xue Li^2,‡^, Pei-Nan Ni^1,*^, Shang-Heng Li^2^, Si-Rui Wang^1^, Wen-Jie Dou^1^, Yong-Yang Zhu^1^, Zhi-Peng Wei^2,*^ and Chong-Xin Shan^1,*^

^1^Henan Key Laboratory of Diamond Materials and Devices, Key Laboratory of Materials Physics, Ministry of Education, School of Physics, Zhengzhou University, Zhengzhou 450052, China

^2^State Key Laboratory of High-Power Semiconductor Lasers, School of Physics, Changchun University of Science and Technology, Changchun 130022, China

‡These authors contributed equally.

* E-mail: [nipeinan@zzu.edu.cn](mailto:nipeinan@zzu.edu.cn); zpweicust@126.com; [cxshan@zzu.edu.cn](mailto:cxshan@zzu.edu.cn)

**Keywords:** diamond, lab-on-a-chip, metasurfaces, optical tweezers, thermal robustness

**Supplementary Note 1:**

**Design of diamond metalenses:**

For the design of diamond metalenses, localized phase delays are imparted to the transmitted light, thereby allowing for wavefront modulation with tailored functionalities. We employed the finite difference time domain method (FDTD) to simulate circular diamond nanopillars with a fixed height but tunable radii. The simulations were conducted at wavelengths of 532 nm and 1064 nm, respectively. In the simulations, periodic boundary conditions were applied along all the in-plane directions, while perfectly matched layer (PML) boundary condition was used in the light propagation direction. The simulation results for the metalens are presented in Fig. S1. Using nanopillars as the base unit, a diamond metalens with dimensions of 200 × 200 μm was designed. The metalens for the 532 nm wavelength features a period of 400 nm and a nanopillars height of 600 nm. To achieve the desired phase profile, eight meta-elements with radii of 50, 65, 73,78, 83, 88, 96, and 107 nm were selected. The metalens for the 1064 nm wavelength features a period of 700 nm and a nanopillars height of 1000 nm. To achieve the desired phase profile, eight meta-elements with radii of 76, 126, 147, 160, 172, 187, 207, and 236 nm were selected. To achieve the focusing functionality, the phase profile of the metalens was designed according to the following formula.

$$\text{ϕ}\text{(}\text{x,y,f}\text{)= 2π -}\frac{\text{2π}}{\text{λ}}\text{ (}\sqrt{\text{x}^{\text{2}}\text{+}\text{y}^{\text{2}}\text{+}\text{f}^{\text{2}}}\text{ - }\text{f}\text{)}$$

where λ is the wavelength of incident light, f is the focus length, and (*x, y*) denotes the coordination of a given point at the metasurface plane.

**Supplementary Note 2:**

**Fabrication of diamond metalenses:**

The fabrication of diamond metasurfaces. First, the double-side polished single-crystal diamond was immersed in aqua regia solution (HCl:HNO₃ = 3:1) for 3 hours to remove metal impurities from its surface. The diamond was then subjected to sequential 15 minutes ultrasonic cleaning in acetone, ethanol, and deionized water to eliminate any organic contaminants. Subsequently, electron beam lithography (EBL) was employed to define the pattern, which was followed by the deposition of a 50 nm thick nickel (Ni) layer via electron beam evaporation to serve as a hard mask. Following the lift-off process, the metal pattern was transferred into the diamond substrate using reactive ion etching (RIE) with oxygen plasma at an RF power of 200 W. Finally, the Ni mask was removed by chemical etching in a 1:2 HCl:HNO₃ solution, thus completing the fabrication of the diamond metasurfaces. In electron-beam lithography, we used PMMA 950 K A4 resist with a thickness of 240 nm and an exposure dose of 250 μC·cm⁻². Flow chart for the fabrication of diamond metasurface is provided in Supplementary Fig. S2.

**Supplementary Note 3:**

**Design of diamond vortex metasurfaces with varying topological charges:**

The diamond metasurface for microparticle rotation operates on the geometric phase principle. The design principle involves modulating the local phase profile, and thus the wavefront, by spatially rotating the orientation angle of each nan-fin under circularly polarized illumination. The simulation setup employed the FDTD method at a wavelength of 532 nm for a nanofin with fixed dimensions (240 nm in length, 115 nm in width, and 600 nm in height, with a lattice period of 500 nm). Periodic and perfectly matched layer (PML) boundary conditions were applied in the in-plane and out-of-plane directions, respectively. The simulation data for this single nanofin is provided in Supplementary Figure S3. Finally, the diamond metasurface was designed as a spiral phase plate according to the following formula.

$$\text{ϕ(}\text{x,y}\text{)=}\text{l}\text{×arctan(}\frac{\text{y}}{\text{x}}\text{)}$$

*l* represents the topological charge of the spiral phase plate. Designs with topological charges of *l*=10,20, and 30 were investigated. The coordinates (*x,y*) denote the position of a given point on the metasurface plane.

**
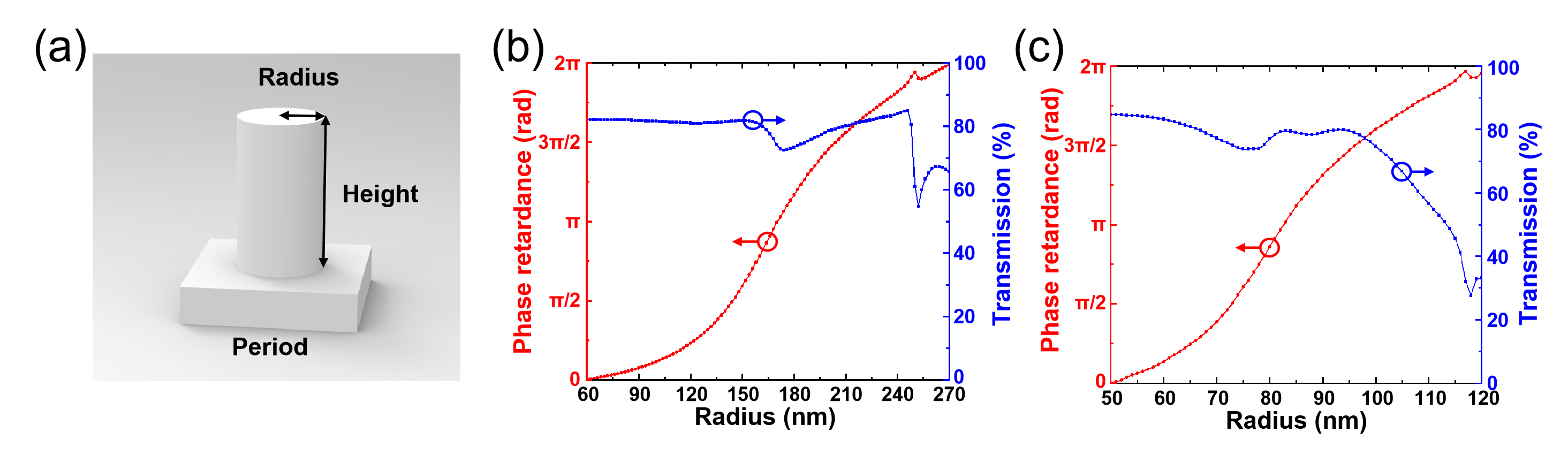
**

**Figure S1.** FDTD simulation of diamond metasurface; (a) Single nanopillar structure diagram; (b) Phase retardance and transmission of the light (λ=1064nm) modulated by diamond nanopillars with varying radius; (c) Phase retardance and transmission of the light (λ=532nm) modulated by diamond nanopillars with varying radius;


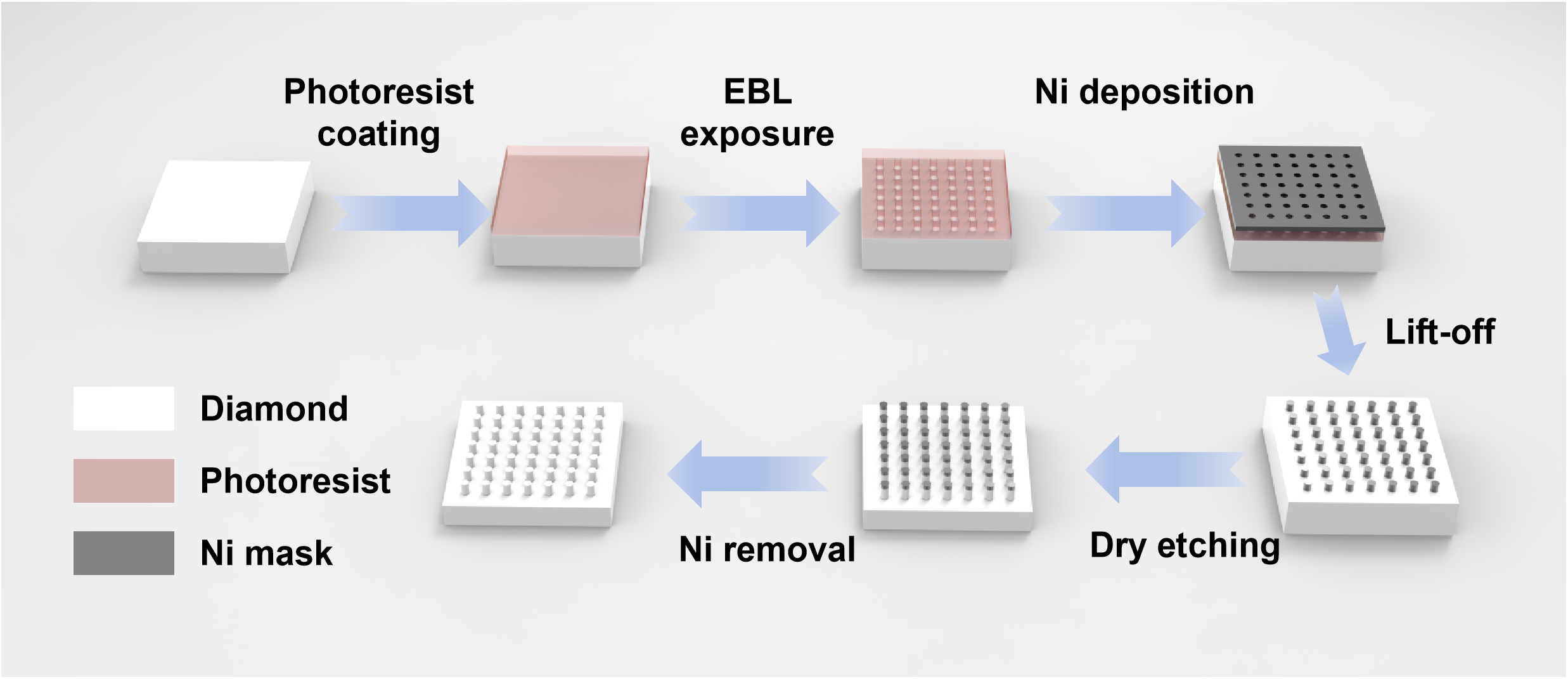


**Figure S2.** The developed nanofabrication processes of diamond metasurfaces.

**
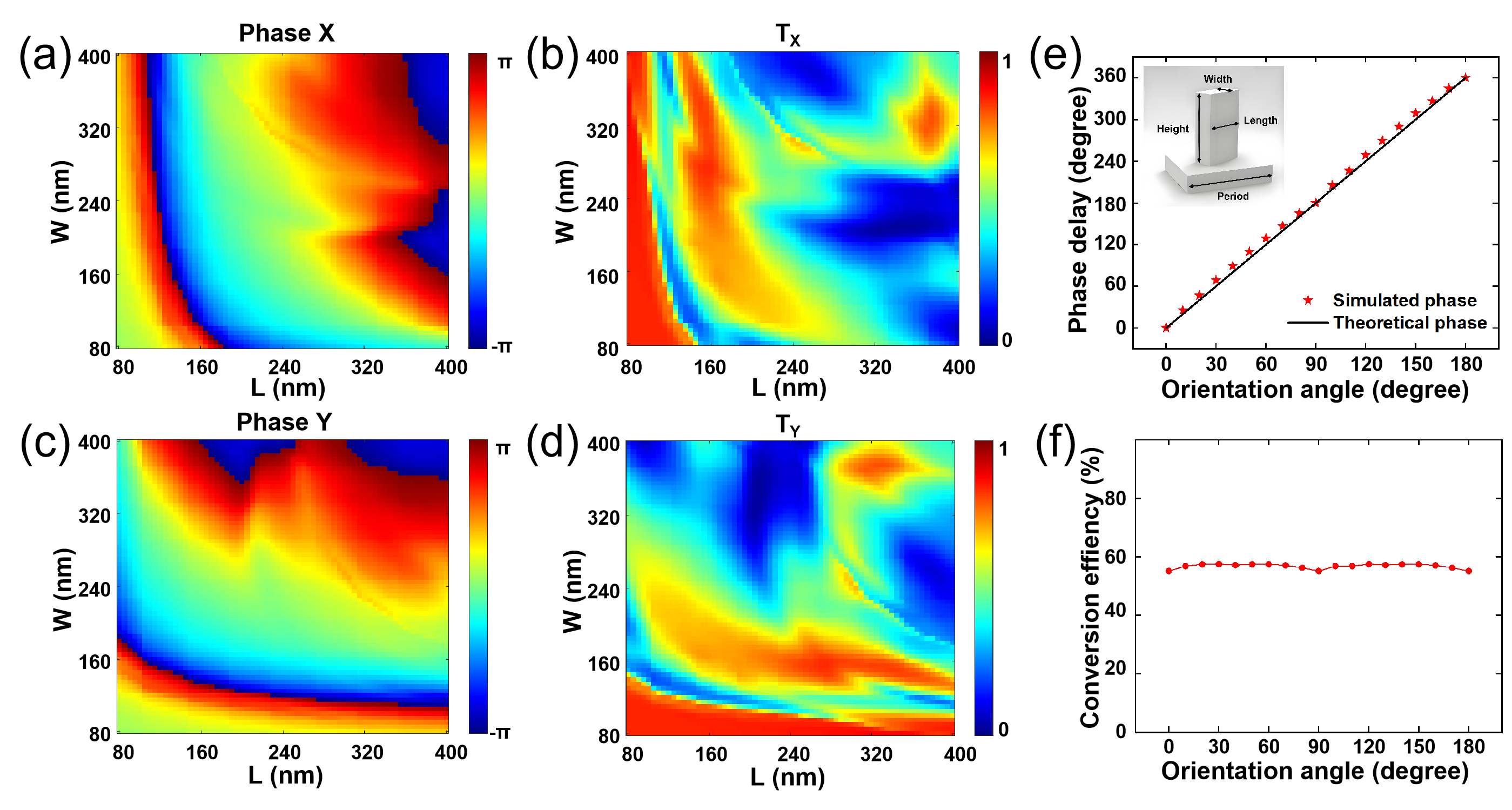
**

**Figure S3.** (a-b) Phase and transmittance modulation of *x*-polarised light at 532 nm wavelength by diamond nanopillars with rectangular cross-section of varying aspect ratios; (c-d) Phase and transmittance modulation of *y-*polarised light at 532 nm wavelength by diamond nanopillars with rectangular cross-section of varying aspect ratios; (e) Phase modulation by diamond nanopillars at different rotation angles, the inset shows the schematic representation of the diamond nanopillars with rectangular shape; (f) Polarization conversion efficiency of diamond nanopillars at different rotation angles.


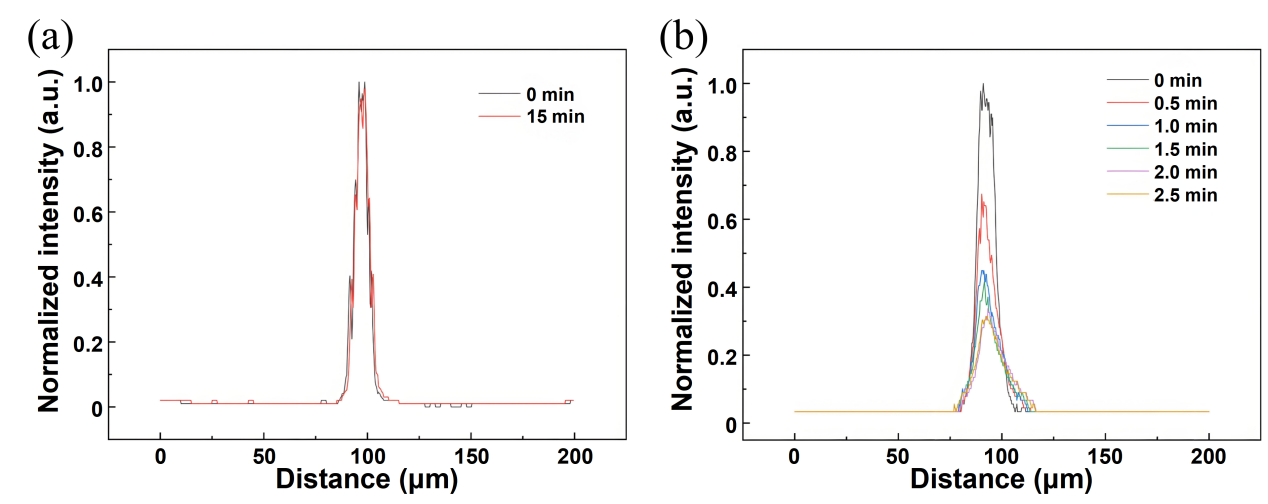


**Figure S4.** Comparison of the temporal stability of the focusing performance for (a) the diamond metalens and (b) a conventional objective lens under continuous 30 W laser illumination. The intensity profile at the original focal plane of the metalens shows almost no change over time, whereas that of the objective lens decays progressively.


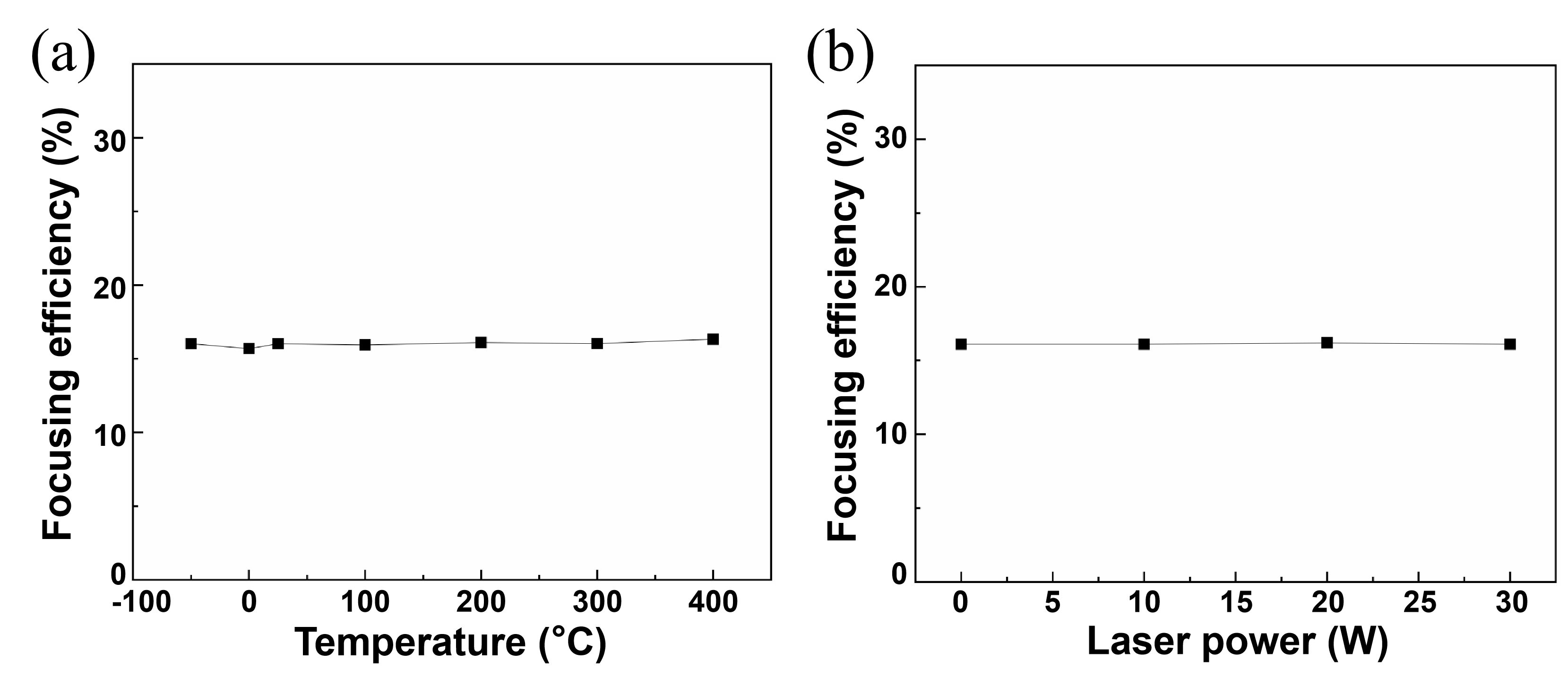


**Figure S5.** (a) Measured focusing efficiency of the diamond metalens across a range of temperatures. (b) Focusing efficiency as a function of incident laser power.

| **Material** | **Coefficients of thermal expansion (K^−1^)** | **Thermo-optic coefficient (K^−1^)** | **Hardness** |
| --- | --- | --- | --- |
| Diamond | 1×10^−6 [1]^ | 6.5×10 ^−6 [2]^ | 10 ^[8]^ |
| Si | 2.7×10^−6 [3]^ | 1.8×10^−4 [4]^ | 6.5 ^[9]^ |
| GaN | 5.59×10^−6 [5]^ | 1.6×10^−4 [5]^ | 7 ^[10]^ |
| TiO_2_ | 2.3×10^−5 [6]^ | -5.0 × 10^−5 [7]^ | 6.2 ^[11]^ |

**Table S1.** Thermal expansion coefficient, thermal optical coefficient and hardness of diamond and other common metasurface materials.

**Reference:**

[1] Jacobson, P. S. S. Thermal expansion coefficient of diamond in a wide temperature range. *Diamond Relat. Mater.* 97 (2019).

[2] Hu, Z., & Hess, P. Optical constants and thermo-optic coefficients of nanocrystalline diamond films at 30–500°C. *Appl. Phys. Lett.* 89, 081906 (2006).

[3] Okada, Y., & Tokumaru, Y. Precise determination of lattice parameter and thermal expansion coefficient of silicon between 300 and 1500 K. *J. Appl. Phys.* 56, 314–320 (1984).

[4] Coenen, D., Kim, M., Oprins, H., et al. A Critical Analysis of the Thermo-Optic Time Constant in Si Photonic Devices. *Photonics* 11, 603 (2024).

[5] Watanabe, N., Kimoto, T., & Suda, J. Thermo-optic coefficients of 4H-SiC, GaN, and AlN for ultraviolet to infrared regions up to 500°C. *Jpn. J. Appl. Phys.* 51, 112101 (2012).

[6] Mashreghi, A. Determining the volume thermal expansion coefficient of TiO₂ nanoparticle by molecular dynamics simulation. *Comput. Mater. Sci.* 62, 60–64 (2012).

[7] Chen, Z., Wei, M., Luo, Y., Jian, J.-H., Ye, Y., Yin, Y., et al. Efficient and compact sol-gel TiO₂ thermo-optic microring resonator modulator.*Opt. Mater. Express.* 12, 4061–4071 (2022).

[8] Vavilov, V. S. The properties of natural and synthetic diamond. Uspekhi Fizicheskih Nauk 163, 99 (1992).

[9] Samsonov, G. V., Ed. Handbook of the Physicochemical Properties of the Elements; IFI/Plenum: New York, 1968.

[10] Dong, Z.; Zhang, X.; Peng, S.; Jin, F.; Wan, Q.; Xue, J.; Yi, X. Mechanical properties of GaN single crystals upon C ion irradiation: Nanoindentation analysis. Materials 15, 1210 (2022).

[11] Lide, D. R., Ed. CRC Handbook of Chemistry and Physics, 84th ed.; CRC Press: Boca Raton, FL, 2004.
